# Supplementary material for: DNA Methylation and Gene Expression in Blood and Adipose Tissue of Adult Offspring of Women with Diabetes in Pregnancy—A Validation Study of DNA Methylation Changes Identified in Adolescent Offspring
Source: Biomedicines. 2022 May 26;10(6):1244. doi: 10.3390/biomedicines10061244 (PMC9219870; doi:10.3390/biomedicines10061244)
Supplement: Supplementary file 1 [file biomedicines-10-01244-s001.zip › biomedicines-1735571-supplementary.pdf]

Table S1. Target genes, respective primer sequences and expected PCR product.

Target Gene	Primer Sequence	Expected Amplification Product
ESM1	F - 5’ GCTACCGCACAGTCTCAGGC 3’	72 bp (2 transcript variants)
	R - 5’ CCCCATTAGAAGGCTGACACC 3’	
MS4A3	F - 5’ GACTTGGGAGGAAAGCCGT 3’	91 bp, 262 bp, and 400 bp (3 transcript variants)
	R - 5’ TGTGGGTTTTATCCCTGCTACA 3’	
TSPAN14	F - 5’ GTCCCGTCTTCCTATCGCTG 3’	79 bp (several transcript variants)
	R - 5’ GGAAAGAAAGGGAGGGAGCC 3’	
TFIIB	F – 5’ GTTCTGTTCCAACCTTTGTCT 3’	51 bp (2 transcript variants)
	R – 5’ TGTGTAGCTGCCATCTGTACTT 3’	
***ESM1* pyrosequencing**	F – 5´ GGTATTTGGAGATGGTAATATTGTGAG 3’	260 bp
	R – 5´ TCCACCATACATCACATTTAATCTT 3’	
	Seq – 5´ GAAGAAGTTGTGAAAGAG 3’	
***MS4A3* pyrosequencing**	F – 5´AGTAGTTGTTTTAGGGTTTAGGTGTTA 3’	225 bp
	R – 5´ CCCCTAATCCATACCCCAAAA 3’	
	Seq – 5´AAAAAAACCAAATCCTAATCCTACA 3’	
***TSPAN14* pyrosequencing**	F – 5´TTTTATTAAGGAGGAAGGGGGAAGT 3’	85 bp
	R – 5´CTTTAACCTCATTTAACCCAACCCTATA 3’	
	Seq – 5´AGTTTGGTTTTTTTTATTGTTT 3’	

Table S2. Baseline data of mothers (1978-85)

	GDM	T1D	BP	GDM vs. BP *P-value*	T1D vs. BP *P-value*
	82	67	57		
**Maternal data (1978-85)**					
Age at delivery (years)	30.39 (5.16)	26.40 (4.67)	26.79 (4.61)	**<0.0001**	0.645
Nordic Caucasian ethnicity (yes vs. no)	94% (77/82)	97% (65/67)	93% (53/57)	0.828	0.297
Pregestational BMI (kg/m^2)	24.28 (5.60)	21.74 (1.93)	21.20 (3.47)	**<0.0001**	0.301
Pre-gestational overweight (BMI ≥ 25 kg/m^2)	34% (28/82)	8% (5/64)	12% (7/57)	**0.003**	0.412
Family history of diabetes (yes vs. no)	26% (21/82)	25% (17/67)	16% (9/57)	0.166	0.191
Fasting Blood glucose before OGTT (mmol/l)	5.2 (0.6)	NA	NA		
120 min blood glucose during OGTT (mmol/l)	7.9 (1.8)	NA	NA		
Mean blood glucose in first trimester	NA	9.0 (3.0)	NA		
Mean blood glucose in third trimester	NA	6.7 (1.6)	NA		

Data are mean (SD) or percentage (number). Analysis of differences between the two groups were performed by independent samples t-test, Mann-Whitney U test or $\chi 2$, respectively. *P*-values < 0.05 are bold. Abbreviations: BMI: body mass index; NA: not available; BP: background population; GDM: gestational diabetes; OGTT: oral glucose tolerance test; T1D: type 1 diabetes.

Table S3. Association between DNA methylation of *ESM1*, *TSPAN14* and *MS4A3*, and maternal diabetes in pregnancy and pre-pregnancy BMI.

**O-GDM compared to O-BP:**	***Association to Group (O-GDM vs. O-BP)***		***Association to Maternal pre-pregnancy BMI***	
	β (95% CI)	*p*-value	β (95% CI)	*p*-value
**Blood DNA methylation (n=139)**				
ESM1 (cg00992687)	-1.1 (-5.1, 2.8)	*0.57*	-0.2 (-0.5, 0.2)	*0.44*
ESM1 (CpG 2)	-0.3 (-4.8, 4.1)	*0.87*	-0.2 (-0.6, 0.2)	*0.40*
ESM1 (CpG 3)	1.0 (-1.4, 3.4)	*0.41*	-0.2 (-0.4, 0.1)	*0.20*
ESM1 (cg09452568)	0.3 (-2.8, 3.5)	*0.85*	-0.1 (-0.5, 0.2)	*0.36*
TSPAN14 (cg11411705)	-0.4 (-1.8, 1.0)	*0.59*	-0.1 (-0.2, 0.9)	*0.49*
MS4A3 (cg14328641)	0.3 (-1.6, 2.2)	*0.74*	-0.1 (-0.3, 0.1)	*0.31*
MS4A3 (CpG 2)	-0.1 (-1.9, 1.7)	*0.91*	0.1 (-0.1, 0.2)	*0.54*
**SAT DNA methylation (n=83)**				
ESM1 (cg00992687)	0.6 (-1.4, 2.5)	*0.57*	-0.1 (-0.2, 0.1)	*0.62*
ESM1 (CpG 2)	-0.4 (-2.2, 1.4)	*0.63*	0.1 (-0.1, 0.3)	*0.25*
ESM1 (CpG 3)	-0.1 (-3.2, 3.1)	*0.97*	0.2 (-0.1, 0.5)	*0.20*
ESM1 (cg09452568)	0.2 (-1.4, 1.9)	*0.80*	0.1 (-0.1, 0.3)	*0.26*
TSPAN14 (cg11411705)	0.02 (-1.6,1.7)	*0.98*	-0.1 (-0.3, 0.1)	*0.18*
MS4A3 (cg14328641)	1.1 (-1.0, 3.2)	*0.30*	-0.1 (-0.3, 0.1)	*0.26*
MS4A3 (CpG 2)	1.2 (-0.1, 2.5)	*0.08*	-0.1 (-0.2, 0.1)	*0.39*

**O-T1D compared to O-BP:**	***Association to Group (O-T1D vs. O-BP)***		***Association to Maternal pre-pregnancy BMI***	
	β (95% CI)	*p*-value	β (95% CI)	*p*-value
**Blood DNA methylation (n=121)**				
ESM1 (cg00992687)	-0.1 (-4.0, 3.8)	*0.97*	-0.1 (-0.8, 0.6)	*0.74*
ESM1 (CpG 2)	0.5 (-3.9, 4.9)	*0.82*	0.1 (-0.7, 0.9)	*0.73*
ESM1 (CpG 3)	0.1 (-2.2, 2.4)	*0.94*	-0.1 (-0.6, 0.3)	*0.50*
ESM1 (cg09452568)	-0.2 (-3.5, 3.0)	*0.88*	0.1 (-0.5, 0.7)	*0.79*
TSPAN14 (cg11411705)	-0.3 (-1.6, 1.0)	*0.70*	0.01 (-0.2, 0.2)	*0.98*
MS4A3 (cg14328641)	0.8 (-1.0, 2.5)	*0.40*	0.1 (-0.2, 0.4)	*0.46*
MS4A3 (CpG 2)	0.4 (-1.5, 2.2)	*0.71*	0.1 (-0.2, 0.4)	*0.64*
**SAT DNA methylation (n=83)**				
ESM1 (cg00992687)	1.0 (-0.5, 2.7)	*0.18*	-0.3 (-0.6, -0.01)	***0.04***
ESM1 (CpG 2)	0.3 (-1.2, 1.8)	*0.70*	-0.1 (-0.3, 0.2)	*0.82*
ESM1 (CpG 3)	0.1 (-3.3, 3.4)	*0.98*	0.1 (-0.6, 0.7)	*0.86*
ESM1 (cg09452568)	1.5 (-0.1, 3.1)	*0.051*	0.1 (-0.2, 0.4)	*0.44*
TSPAN14 (cg11411705)	0.9 (-1.0, 2.8)	*0.35*	-0.2 (-0.6, 0.2)	*0.28*
MS4A3 (cg14328641)	1.9 (-0.1, 3.9)	*0.06*	-0.1 (-0.4, 0.4)	*0.96*
MS4A3 (CpG 2)	1.8 (0.4, 3.2)	***0.01***	0.1 (-0.2, 0.4)	*0.43*

Estimated difference in DNA methylation percentage between O-BP and O-GDM/O-T1D is presented as β (95% CI) and *p*-value, as calculated in the regression model including the covariates of maternal diabetes status, pre-pregnancy maternal BMI (continuous variable, per BMI unit) and offspring sex. O-GDM: offspring of women with GDM, O-T1D: offspring of women with T1D, O-BP: offspring of women from the background population, SAT: subcutaneous adipose tissue, mBMI: maternal pre-gestational BMI.

Table S4. Correlations between SAT *ESM1* and *TSPAN14* expression and offspring clinical characteristics.

	***ESM1* SAT expression**		
	O-GDM (n=22-23)	O-T1D (n=41-42)	O-BP (n=26)
**Diastolic BP (mmHg)**	**-0.53 (0.01)**	-0.09 (0.55)	-0.14 (0.51)
**HbA1c (mmol/mol)**	-0.20 (0.36)	-0.08 (0.64)	-0.03 (0.89)
**Fat (%)**	-0.21 (0.35)	0.10 (0.52)	0.32 (0.11)
	***TSPAN14* SAT expression**		
	O-GDM (n=57-58)	O-T1D (n=60)	O-BP (n=42-57)
**Diastolic BP (mmHg)**	0.19 (0.15)	0.05 (0.68)	0.21 (0.19)
**HbA1c (mmol/mol)**	**0.29 (0.03)**	-0.10 (0.45)	0.21 (0.18)
**Fat (%)**	**0.34 (0.01)**	0.10 (0.44)	0.04 (0.80)

r coefficient (*p*-value). O-GDM: offspring of women with GDM, O-T1D: offspring of women with T1D, O-BP: offspring of women from the background population; SAT: subcutaneous adipose tissue, BP: blood pressure. The correlation analysis was performed via Spearman rank correlation test. *P*-values <0.05 are presented in bold.
